# Cutting edge technologies expose the temporal regulation of neurogenesis in the *Drosophila* nervous system

**DOI:** 10.1080/19336934.2022.2073158

**Published:** 2022-05-13

**Authors:** Makoto Sato, Takumi Suzuki

**Affiliations:** a Mathematical Neuroscience Unit, Institute for Frontier Science Initiative,Laboratory of Developmental Neurobiology, Graduate School of Medical Sciences, Kanazawa University, Ishikawa, Japan; b College of Science, Department of Science, Ibaraki University, Ibaraki, Japan

**Keywords:** Drosophila, neural development, temporal patterning

## Abstract

During the development of the central nervous system (CNS), extremely large numbers of neurons are produced in a regular fashion to form precise neural circuits. During this process, neural progenitor cells produce different neurons over time due to their intrinsic gene regulatory mechanisms as well as extrinsic mechanisms. The *Drosophila* CNS has played an important role in elucidating the temporal mechanisms that control neurogenesis over time. It has been shown that a series of temporal transcription factors are sequentially expressed in neural progenitor cells and regulate the temporal specification of neurons in the embryonic CNS. Additionally, similar mechanisms are found in the developing optic lobe and central brain in the larval CNS. However, it is difficult to elucidate the function of numerous molecules in many different cell types solely by molecular genetic approaches. Recently, omics analysis using single-cell RNA-seq and other methods has been used to study the *Drosophila* nervous system on a large scale and is making a significant contribution to the understanding of the temporal mechanisms of neurogenesis. In this article, recent findings on the temporal patterning of neurogenesis and the contributions of cutting-edge technologies will be reviewed.

## Introduction

During the development of the brain in various animals, extremely large numbers of neurons are produced to form precise neural circuits. The process of producing various neurons from neural progenitors is precisely controlled both spatially and temporally [1–5]. Spatial control is achieved by controlling the properties of neural progenitors and neurons according to pattern formation mechanisms, such as positional information provided by morphogens. Temporal regulation, on the other hand, is controlled by a series of temporally-expressed transcription factors or temporal gradients of RNA-binding proteins in neural progenitors, and epigenetic mechanisms also play important roles in this process.

**Figure 1.**
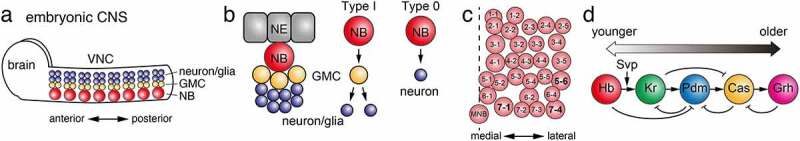
Temporal patterning in the embryonic CNS (a) NBs in the VNC and brain in the embryonic CNS produces GMCs and neurons and/or glial cells. (b) During embryonic CNS development, a NB, differentiated from a NE cell, is delaminated from the embryonic epithelia. A Type I NB produces multiple GMCs through asymmetric cell divisions. A GMC divides to produce two cells that give rise to either neuron or glial cell. A Type 0 NB directly produces nerons. (c) Spatial distribution of NBs in an embryonic hemisegment. A broken line indicates the midline. MNB, midline neuroblast. (d) A series of temporal transcription factors, Hb, Kr, Pdm, Cas and Grh, are sequentially expressed in a NB due to the feed-forward and feed-back gene regulatory network. Additionally, Svp regulates the transition from Hb to Kr expression.

*Drosophila melanogaster* has been used as a model system for a variety of biological phenomena, but it has played a particularly important role in elucidating the mechanisms of time-dependent regulation of neural diversity. In the embryonic central nervous system (CNS), a series of temporal transcription factors (TTFs) are sequentially expressed over time in neural progenitor cells called neuroblasts (NBs; Figure 1) [6]. Although NBs in embryonic CNS enter quiescent period at the end of embryogenesis, they are reactivated in the beginning of larval development in a nutrient dependent manner [7,8]. These larval NBs produce a wide variety of neurons that establish neural circuits in the adult CNS [9].

**Figure 2.**
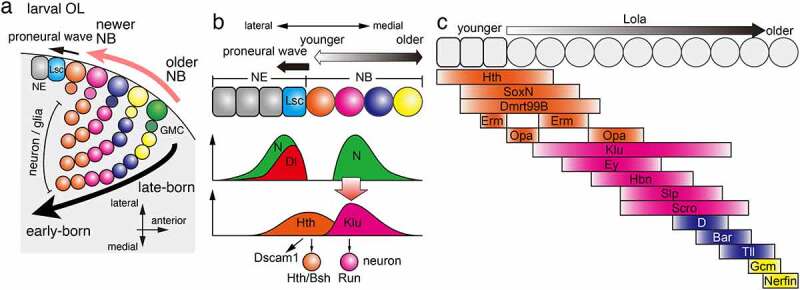
Temporal patterning in the optic lobe (a) During medulla development in the OL, NBs are sequentially differentiated from NEs behind the proneural wave, which is marked by the expression of Lsc. A NB produce a GMC, which divides to produce neurons and/or glial cells inside the brain. Sequential TTF expression in NBs enables sequential production of different types of cells in the brain. (b) Hth expressed in NEs and the youngest NBs triggers the expression of Dscam1 and the production of Hth/Bsh-positive neurons. Dl expressed at the proneural wave front activates Notch signalling at the wave front and NBs distant from the wave front. The Notch activity in NBs triggers the expression of Klu and the production of Run-positive neurons. (c) Multiple temporal transcription factors are sequentially expressed in NEs and NBs. Erm and Opa are expressed twice forming a gap between the two expression domains. Lola is expressed throughout NBs to control the speed of the temporal cascade progression.

During development of the medulla in the larval optic lobe (OL), NBs are produced row by row on the surface of the brain, following a wave of differentiation called the proneural wave (Figure 2) [10–13]. Here, a completely different group of TTFs is expressed in NBs than in the embryonic CNS, and different types of neurons are produced towards the inner side of the brain over time [14,15]. This results in the formation of a concentric pattern, with early-born neurons located deep inside the brain (Figure 2a) [16,17]. The lobula and lobula plate of the optic lobe also produce a variety of neurons in a time-dependent manner [18].

**Figure 3.**
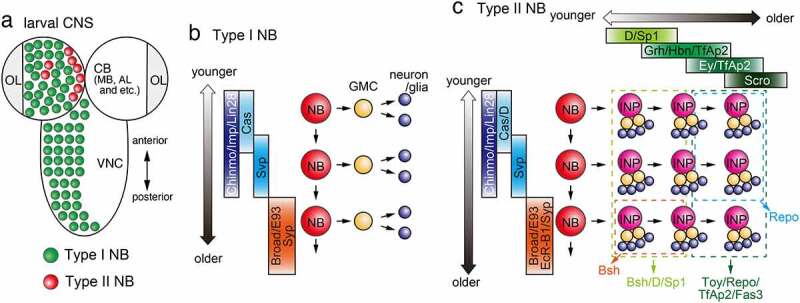
Temporal patterning in the central brain (a) Type I and Type II NBs in the CB and Type I NBs in the VNC in the larval CNS. NBs in the OL are not shown. (b) In the developing central brain, a Type I NB sequentially differentiate over time and produce multiple GMCs and neurons (and/or glial cells). A Type I NB sequentially expresses Cas, Svp, Chinmo/Imp/Lin28 and Broad/E93/Syp. (c) A Type II NB sequentially expresses Cas/D, Svp, Chinmo/Imp/Lin28 and Broad/E93/EcR-B1/Syp producing multiple INPs. An INP then sequentially expresses D/Sp1, Grh/Hbn/TfAp2, Ey/TfAp2 and Scro. Younger INPs and their progeny tend to express Bsh, D and Sp1 (light green box). Older INPs and their progeny tend to express Toy, Repo, TfAp2 and Fas3 (dark green box). Bsh-positive neurons are produced from younger INPs that derive from older NBs (Orange box). Repo-positive glial cells are produced from older INPs that derive from younger NBs (light blue box).

During larval central brain (CB) development, in addition to Type I NBs, which are similar to NBs found in the embryonic CNS and medulla in the OL, there are Type II NBs, which produce the transit amplifying neural progenitors called intermediate neural progenitors (INPs), which undergo limited rounds of asymmetric division to produce GMCs and neurons (Figure 3). In Type II NBs, the expression of TTFs changes over time not only in the NBs but also in the INPs generated from Type II NBs, which produce a greater variety of neurons than Type I NBs (Figure 3b, c) [19,20]. The NBs in the embryonic CNS, larval VNC and medulla in the larval OL are considered as Type I NBs.

Genetic techniques of *Drosophila* have been used to promote these studies by identifying various TTFs and their interactions. However, it is unlikely that all transcription factors can be identified by such classical methods, and furthermore, non-transcription factors such as noncoding RNAs may also be involved. Recently, next-generation sequencing technologies such as single-cell RNA-seq (scRNA-seq) have made it possible to obtain gene expression profiles of all cells that constitute the nervous system [21–29]. This has led to the identification of a large number of factors that have not been identified using conventional methods and has further advanced our understanding of the mechanisms that regulate neurogenesis in a time-dependent manner. In this review, we will focus on the embryonic CNS, the medulla in the larval OL, and the larval CB and VNC, and review the temporal mechanisms of neurogenesis in each system and the latest findings based on cutting edge technologies.

## Temporal patterning in embryonic CNS neuroblasts

The ventral nerve cord (VNC) of the embryo is considered to be the organ equivalent of the mammalian spinal cord and is the region where the concept of temporal patterning of neurogenesis was first discovered [6,30]. In the VNC of the embryonic CNS, NBs delaminate from the neuroectoderm and are arranged in a regular matrix of 30 cells in each hemisegment (Figure 1a-c) [31,32]. Pioneering work by the Doe lab has shown that Hunchback (Hb), Krüppel (Kr), Nubbin and Pdm2 (Pdm), and Castor (Cas) are each transiently expressed in NBs (Figure 1d) [6,30]. In addition, after Cas, grainy head (Grh) was found to be expressed in NBs [33].

Functional analysis of these TTFs has focused on NB7-1, which generates five different motor neurons U1-U5 in the first five cell divisions (Figure 1c). Hb and Kr are early expressed TTFs that are necessary and sufficient for the fate of early motor neurons [6]. The competence of embryonic NB7-1 (and NB3-1) to produce motoneurons under the control of Kr are restricted by Polycomb repressor complex (PRC) [34]. Thus, the functions of TTFs may be influenced by the epigenetic state of NBs. Pdm is necessary and sufficient for the fate of U4 motor neurons in NB7-1 and, together with Cas, is involved in the fate of U5 motor neurons [35].

The role of Cas and Grh has been analysed in detail in another lineage, NB5-6 (Figure 1c) [36]. In this lineage, Cas is expressed in NBs until the end of embryogenesis, and different types of neurons are produced during the period of Cas expression. In the last four cell divisions of NB5-6, Cas induces the expression of Collier/Knot and Squeeze, thereby specifying Apterous-positive neurons. Thus, temporal patterning generated by TTFs is further subdivided by subtemporal factors [36,37]. Grh has been shown to be required for the fate of late-producing FMRFamide-positive neurons [36].

While a Type I NB produces a GMC that divides once to generate daughter cells, it is transformed to a Type 0 NB that directly produces neurons in the embryonic CNS (Figure 1b) [38]. The Type I > 0 switch is triggered by Dacapo, an evolutionarily conserved cell cycle inhibitor, under the control of Cas and Antennapedia, one of the Hox genes. The following cell cycle exit is regulated by Grh in addition to Cas and Antennapedia.

The molecular mechanism that switches the expression of these TTFs remains unclear. Ectopic expression experiments have shown that each gene induces the next TTF to be expressed, and it represses the expression of the previously expressed TTF (Figure 1d). Additionally, it represses the following target of the next TTF. However, the loss of each TTF does not affect the expression of the next TTF, suggesting the existence of independent regulatory mechanisms [6]. It is known that cytokinesis and the expression of seven up (Svp), an orphan nuclear receptor, in NBs are essential for the transition from Hb to Kr in NBs, and the transition to the Kr temporal window occurs when Svp represses the expression of Hb (Figure 1d) [39,40]. Subnuclear genome reorganization is also involved in silencing the expression of Hb [41]. However, the molecular mechanism regulating the transition of Kr, Pdm, Cas and Grh temporal windows remains elusive.

Understanding of the mechanisms that establish the sequential TTF expression through activator and repressor cross-regulations requires theoretical as well as molecular genetic approaches (Figure 1d). A theoretical study suggested that repressor-decay acts as a more robust timer compared with activator-relay mechanism [42]. This prediction was experimentally validated comparing the temporal changes in TTF expression in control and mutant backgrounds.

Dam methyltransferase is an *E. coli*-derived adenine methyltransferase. When a fusion protein of a transcription factor of interest and Dam is expressed, adenine in the 5´-GATC-3´ sequence near the target sequence is methylated by Dam [43]. Targeted DamID (TaDa), a cell type-specific DNA binding protein targeting the Dam fusion protein under the control of the Gal4/UAS system, was developed by the Brand laboratory and used for target identification of transcription factors [44–50]. Using TaDa, the DNA binding pattern of the transcription factor of interest can be quantitatively analysed in terms of adenine methylation frequency. Since adenine methylation occurs only in cells expressing Dam fusion proteins, the analysis can be performed without cell sorting. By PCR amplification of the methylated DNA fragments and deep sequencing, the binding of the transcription factor of interest with the target sequence can be quantified [45].

TaDa was used to analyse how target binding of Hb differs between NBs in the embryonic CNS (Figure 1c, d). A fusion protein of Hb and Dam was specifically expressed in NB5-6 and NB7-4, and their binding to the target chromatin region was compared. Its binding to the gooseberry (Gsb) locus was stronger in the former and weaker in the latter, and correspondingly, Gsb expression was induced only in the former NBs [51]. This result indicates that the state of chromatin is regulated according to spatial information, which modifies the output of Hb.

MicroRNAs (miRNAs) are a type of small noncoding RNA that are involved in various posttranslational controls. On the other hand, long noncoding RNAs (lncRNAs) are also known to be involved in neuronal cell typing. FACS sorting and scRNA-seq analysis of cells expressing NB markers revealed multiple miRNAs and lncRNAs that are expressed specifically and selectively in NBs [21]. Future studies will reveal the potential roles of miRNAs and lncRNAs in the temporal regulation of neurogenesis.

## Temporal patterning following the proneural wave in the larval optic lobe

Among the four parts of the OL, lamina, medulla, lobula, and lobula plate, temporal patterning of NBs has been extensively studied in the medulla [4]. At the beginning of medulla development, neuroepithelial cells (NEs) are arranged in sheets, and only several embryonic NBs are present [52]. Most medulla NBs are produced by the wave of differentiation, proneural wave, starting from the medial edge of the OL adjacent to the CB towards the lateral side of the brain (Figures 2a, 3a). Behind the wave front of the proneural wave, NEs differentiate into NBs row by row (Figure 2a, b) [10]. Thus, NBs located on the medial side are older NBs, while those located on the lateral side are newly differentiated; therefore, each row of NBs has different temporal information. NBs produce multiple neurons towards the centre of the medulla so that the early-born neurons are located in the centre and the later-born neurons are located outside [16].

We and the Desplan lab have identified several TTFs in medulla NBs that are different from the set of TTFs found in embryonic VNC (Figure 2c) [4,12,14,15,37]. In medulla NBs, Homothorax (Hth), Klumpfuss (Klu), Eyeless (Ey), Sloppy paired 1 and 2 (Slp), Dichaete (D), and Tailless (Tll) are sequentially expressed overlapping with each other (Figure 2c). These TTFs are expressed in almost all NBs except those located at the posterior edge of the developing medulla [53]. The molecular mechanism of the switch in the expression of each TTF is unknown for Hth and Klu, but for the other TTFs such as Ey, Slp, and D, it has been shown that the previously expressed TTF induces the expression of the next TTF, and conversely, the latter represses the expression of the previously expressed TTF [14,15].

The inner part of the developing medulla is divided into concentric zones according to the expression of transcription factors, each of which differentiates into one or several different type(s) of neurons: Hth is expressed in the innermost part of the medulla; brain-specific homeobox (Bsh) is expressed in the outer half of the Hth region; Runt (Run) is expressed outside the Hth/Bsh region; and Drifter (Drf) is expressed outside the Run region [12,16]. The role of each TTF was investigated using the expression of these transcription factors as markers [14,15,54]. During the period of Hth expression in medulla NBs, Bsh-positive neurons are produced, and Hth is necessary and sufficient for the production of Bsh-positive neurons (Figure 2b) [15,16]. Ectopic expression of Klu increases the number of Run-positive neurons (Figure 2b), but Klu mutant cells show a tumour-like phenotype precluding us from examining their physiological function. Ey is necessary and sufficient to suppress the production of Run-positive neurons and to induce the production of Drf-positive neurons [14,15]. In contrast, Slp is necessary and sufficient to suppress the production of Drf-positive neurons. Recent studies have shown that Slp and D are required for the production of neurons expressing Sox102F and Ets65A [55]. In addition, it has been reported that the number of glial cells is reduced in mutant clones of either Ey or D in the medulla. Thus, TTFs are involved in the fate determination of glial cells as well as neurons [54].

Temporal patterning of neural development is important not only for cell type specification but also for neural circuit formation [11]. Indeed, Hth temporally upregulates the transcription of the Down syndrome cell adhesion molecule, Dscam1 (Figure 2b) [56]. The *Drosophila Dscam1* gene has three alternative exons and produces as many as 20,000 different ectodomains. Only identical Dscam1 isoforms can bind with each other and produce a repulsive signal. As a result, neurons expressing the same Dscam1 isoforms repel each other [57–59]. Its temporal transcription in Hth-positive NBs and Dscam1 protein accumulation along the axons of their daughter neurons cause axonal repulsion between neurons of the same lineage. This process, lineage-dependent repulsion, is essential for the formation of the columnar structure, a structural and functional unit of the brain [60]. Thus, Hth regulates column formation through the temporal regulation of Dscam1 expression.

Among the temporal transcription factors in medulla NBs, the mechanism of the transition through Hth, Klu and Ey remains elusive. No genetic interaction has been found between the three factors [14,15]. On the other hand, proneural wave progression is positively and negatively controlled by EGF and Notch signalling, respectively [13,61]. Although the Notch signal is activated at the wave front of the proneural wave to repress its progression, it is activated again in NBs behind the proneural wave. Interestingly, the second peak of Notch activation coincides with Klu expression and upregulates its expression (Figure 2b) [62]. Similarly, Notch signalling controls the expression of Slp in medulla NBs [50]. The temporal dynamics of Notch signal activity may control the temporal regulation of neurogenesis in other developmental contexts.

Several scRNA-seq analyses have been performed in the OL, revealing gene expression profiles in a large number of neurons [22–26,63]. In addition, scRNA-seq analysis has been performed focusing on the developing OL, which has led to a better understanding of the temporal patterning of neurogenesis [27,29]. As a result, many TTFs were newly identified in medulla NBs, and their regulatory network was revealed.

SoxN and Dmrt99B are expressed in NEs and younger NBs overlapping with the Hth domain and are required for the production of Bsh-positive neurons (Figure 2c) [27]. While Hth and SoxN are not required to turn on the expression of later TTFs, Dmrt99B turns on the expression of a subsequent TTF, Odd-paired (Opa), which then represses the previous TTF, Hth.

Earmuff (Erm; ortholog of mammalian Fezf2) and Opa show striped expression patterns with two expression domains. Erm expression domains in NEs and NBs are separated with a gap found in the youngest NBs, where the first Opa domain is found (Figure 2c) [27]. The second Opa domain is found in NBs subsequent to the second Erm domain. The first Opa stripe is required for the repression of the previous TTF, Hth, and activation of the subsequent TTFs, Ey and Erm. On the other hand, the second Erm peak represses Opa expression to form its gap.

Klu was suggested to control the production of Run-positive neurons based on the results of its overexpression experiment. However, its loss of function caused overproliferation of NBs, masking its involvement in Run-positive neuron production [15]. In contrast, Opa was shown to be necessary for the production of Run-positive neurons in a loss-of-function experiment [27]. It is possible to speculate that Klu and Opa act together to control the production of Run-positive neurons.

Hbn and Scro were found to be expressed in the regions of Ey- and Slp-positive NBs. They promote the transition from Ey- to Slp-positive NBs [27,29]. These TTFs have been identified as target genes of Ey by TaDa analysis using the Ey-Dam fusion protein [50].

Two redundant homeobox transcription factors, BarH1 and BarH2 (collectively Bar), are expressed in NBs between the D and Tll domains. D promotes Bar expression, while Bar represses D and promotes Tll expression, suggesting that D, Bar and Tll form a gene regulatory network that controls their sequential transition in NBs [27]. Bar promotes Glial cells missing (Gcm) as well as Tll expression. Gcm then represses Tll expression and promote gliogenesis and cell cycle exit through Dacapo expression. Together with Gcm, Nerfin is also required in the oldest NBs to promote gliogenesis and cell cycle exit.

Finally, Lola is uniformly expressed in the medulla NBs. In the absence of Lola, the temporal progression of TTF expression becomes slower in NBs. Thus, Lola may belong to a unique type of TTF that controls the speed of temporal cascade progression [27].

## Temporal patterning in neuroblasts in the larval CNS

Besides Type II NBs, there are Type I NBs in the larval CB including mushroom body (MB) and antennal lobe (AL). Additionally, there are multiple Type I NBs in the larval VNC (Figure 3a). Robotic sorting of Type I NBs in MB and AL and Type II NB in CB were combined with scRNA-seq analysis to reveal the difference of temporal patterning mechanisms among different NBs [64]. The difference and similarity between Type I and Type II NBs in the larval CNS (CB and VNC) should be unveiled in the future.

It was revealed that the temporal pattering of Type I and Type II NBs in the larval CNS is established by a unique mechanism that involve the expression of two RNA binding proteins: IGF-II mRNA binding protein (Imp) and Syncrip (Syp) (Figure 3b, c) [65,66]. Imp is highly expressed in NBs during early larval stages and is gradually decreased as they age. In contrast, Syp expression gradually increases over time and facilitates termination of neurogenesis [67,68]. This transition from Imp+ to Syp+ period is indispensable for the competence of NBs to respond to ecdysone released from the ring gland during early pupal stage to terminate neurogenesis [67,69].

In addition to Imp and Syp, Chronologically inappropriate morphogenesis (Chinmo), Lin-28, Broad complex (Br), and Ecdysone-induced protein 93 F (E93) are temporally expressed in Type I and Type II NBs of larval CB and VNC (Figure 3b, c) [66,70,71]. Chinmo and Lin-28 are found in Imp+ NBs during early larval stage, while Br and E93 appear in Syp+ NBs during late larval stage. Cas and Svp also appear in the NBs in larval CNS prior to the beginning of Imp/Chinmo/Lin-28 expression [70,72]. While Hb/Pdm/Cas have no role in an AL Type I NB lineage (adPN), Kr is required for one temporal fate during embryonic stage, while Cas is required for multiple temporal fates in later embryonic and larval stages [73].

How is the temporal transition from Imp/Chinmo/Lin-28 to Syn/Br/E93 regulated? In the absence of Svp, Imp/Chinmo/Lin-28 expression is maintained in the NBs even during late larval stage [70]. Ecdysone is released from a ring gland and control temporal progression of the NBs from early to late temporal window cell non-autonomously. Chinmo expression is maintained in Type I and Type II NBs during late larval stage in *ecdysoneless* mutant larvae, which show lowered level of haemolymph ecdysone titre [71]. The temporal action of Ecdysone and temporal gradients of proteins have been reported in Type I NBs in MB [74–76] and Type II NBs in CB [64,71,77], and may be one of general mechanisms to produce diverse neurons in the *Drosophila* CNS. Although some clues have been found, molecular mechanisms that regulate the temporal transition from Imp/Chinmo/Lin-28 to Syn/Br/E93 period are largely unknown.

A secreted signalling molecule, Hedgehog (Hh), is also involved in the temporal patterning of Type I NBs in the larval CB by promoting their cell cycle exit [78]. Hh expression is promoted by Cas and Hh acts as an autocrine or paracrine to regulate the cell cycle exit of NBs together with Grh. Temporal regulation of neural progenitors by non-autonomous feed back signalling from postmitotic neurons remains to be examined in the *Drosophila* CNS [79].

As demonstrated in the embryonic NBs, the functions TTFs may be influenced by the epigenetic state of NBs [34]. Using TaDa technique, epigenetic states of chromatin were systematically analysed in larval NBs, GMCs and neurons [80]. In combination with molecular genetic studies, similar approaches may reveal the interplay between TTFs and epigenetic mechanisms during temporal patterning of NBs.

Changes in energy metabolism during development is one of the key events to govern temporal progression in the NBs [66,69,81]. The NBs mainly gain energy via glycolytic pathway during larval stages, while they utilize oxidative phosphorylation to produce more energy in pupal stages [66,69]. Although the larval NBs partially rely on oxidative phosphorylation for energy production, the metabolic pathway is still important for successful temporal patterning and consequent initiation of the terminal phase of NB proliferation [81]. Syp, one of the late temporal factors, is involved in the regulation of metabolic genes [66], suggesting that bidirectional interactions exist between temporal factors and metabolic pathways.

## Temporal patterning of neuroblasts and intermediate progenitors in the larval central brain

The dorso-medial (DM1-DM6) and dorso-lateral (DL1-DL2) NBs of larval CB are called Type II NBs and show a unique division pattern (Figure 3a). These Type II NBs differ from other NBs in that they produce transit amplifying neural progenitors called INPs, which undergo multiple asymmetric divisions to give rise to 4–6 GMCs (Figure 3c) [82–84]. INPs initially give rise to Bsh/D-positive neurons and later produce Twin of eyeless (Toy)-positive neurons and glial cells. Therefore, temporal patterning of neurogenesis occurs in INPs in CB as in Type I NBs in embryonic VNC and medulla. Three TTFs, D, Grh, and Ey, have been identified in CB INPs (Figure 3c) [19]. In the INPs, D is expressed early and switches to Ey expression late, while Grh is expressed late in the D window and early in the Ey window. Loss of function of D or Grh results in the loss of Bsh-positive neurons, which are produced early in development. Similarly, loss of function of Ey causes a decrease in the number of Toy-positive neurons and glial cells produced later in development [19]. Thus, younger INPs and their progenies tend to express D and Bsh, respectively, while the progenies of older INPs tend to express Toy and Repo. In addition, a recent study identified neurons produced by younger INPs (P-EN, P-FN) and neurons produced by older INPs (E-PG, PF-R) in the central complex in the larval CB, and reported that each type of neuron increases and decreases, respectively, in the loss-of-function of Ey, which specifies the late state of INPs [85].

In addition to the mutual regulation between TTFs, two different factors have been identified as regulators of TTF expression by RNAi screening [86]. Osa, a component of SWI/SNF chromatin-remodelling complex, and its target Hamlet are essential for D expression and suppression of Grh in the INPs, respectively.

INPs give rise to different types of cells early and late in development (Figure 3c) [19], suggesting that temporal factors are also expressed in Type II NBs and determine the nature of the INPs they produce. In fact, it has been reported that Cas/D is initially expressed in younger Type II NBs, followed by the switch to Svp expression (Figure 3c) [19]. Bsh-positive neurons are produced from younger INPs derived from older NBs, while Repo-positive glial cells are produced from older INPs derived from younger NBs.

The transcriptome analysis of Type II NBs was performed using the TU-tagging method developed by the Doe lab, and several temporal factors were identified [71]. First, Type II NBs can be divided into early stage, when Cas, Svp, Chinmo, Imp and Lin28 are expressed, and late stage, when Ecdysone receptor B1 (EcR-B1), Syp, Broad and E93 are expressed (Figure 3c). Svp, one of the early TTFs, is necessary for the expression of the late temporal factors EcR-B1, Syp, Broad and E93 and for the suppression of Chinmo and Imp expression. Importantly, EcRB1 acts as an ecdysone receptor and controls target gene transcription. Ecdysone has been recognized as an exogenous factor that regulates temporal patterning in type II NBs [71,77].

Compared with the embryonic CNS and OL, relatively fewer TTFs have been identified in Type II NBs in the CB. Type II NBs were specifically labelled and FACS sorted to perform Type II NB-specific scRNA-seq analyses [28]. The pseudotime analysis confirmed the temporal cascade of TTFs in INPs proposed in the previous genetic studies [19,20]. Furthermore, additional temporal factors were identified. Sp1 is expressed in the D-positive younger INPs, which produce Bsh-, D- and Sp1-positive progenies (Figure 3c). TfAp2 is expressed in Grh- and Ey-positive older INPs, which produce Toy-, TfAp2- and Fas3-positive neurons and Repo-positive glial cells. Further genetic analysis is necessary to reveal the gene regulatory network between these TTFs.

scRNA-seq analyses only provide RNA expression profiles. TaDa may be a powerful approach that identifies direct transcriptional targets of TTFs. NanoDam is an improved version of TaDa, which enables TaDa analysis for endogenously GFP-tagged proteins without generating a Dam fusion construct, and can be used to search for new TTFs [87]. By using a fusion protein of anti-GFP antibody and Dam that binds with the GFP-tagged transcription factor, the transcriptional targets of TTFs can be efficiently and systematically identified. Using this method, transcriptional targets of D, Grh and Ey were investigated, and the results were compared with the scRNA-seq dataset to identify new TTFs in INPs such as Hbn and Scro.

Hbn is a target of D, Grh and Ey according to the TaDa analysis and is expressed in Grh-positive middle-aged INPs (Figure 3c). Hbn activates the subsequent TTF, Ey, which then terminates Hbn expression in the older INPs. Scro is expressed in the oldest INPs. Consistent with the results of TaDa analysis, Scro is a target of Ey and is upregulated by Ey. Scro represses Ey expression to promote the transition to the oldest temporal window of INPs. Interestingly, Hbn and Scro similarly act as TTFs in the medulla NBs (Figure 2c) [27,29,87].

## Data Availability

The authors declare that there is no data to be shared.

## Future perspectives

scRNA-seq analysis is very powerful, and can identify many unknown factors and provide many insights into the gene regulatory network. Noncoding genes such as miRNAs and lncRNAs may also play important roles in the temporal cascade of neurogenesis. Combined with techniques such as TaDa and NanoDam, scRNA-seq analysis can systematically reveal direct targets of TTFs. Ultimately, however, direct confirmation by genetic approaches, which requires significant efforts, is necessary. Further integration with innovative technologies will be required to elucidate the complex mechanisms of the temporal cascade in a wide variety of cell types, including Type I NBs, Type II NBs and INPs.
